# PACAP and Other Neuropeptide Targets Link Chronic Migraine and Opioid-induced Hyperalgesia in Mouse Models*

**DOI:** 10.1074/mcp.RA119.001767

**Published:** 2020-10-10

**Authors:** Krishna D.B. Anapindi, Ning Yang, Elena V. Romanova, Stanislav S. Rubakhin, Alycia Tipton, Isaac Dripps, Zoie Sheets, Jonathan V. Sweedler, Amynah A. Pradhan

**Affiliations:** ‡ Department of Chemistry, University of Illinois at Urbana-Champaign, 61801; § Beckman Institute for Advanced Science and Technology, University of Illinois at Urbana-Champaign, 61801; ¶ Department of Psychiatry, University of Illinois at Chicago, 60612

## Abstract

Chronic use of opioids can produce opioid-induced hyperalgesia (OIH), and when used to treat migraine, these drugs can result in increased pain and headache chronicity. We hypothesized that overlapping mechanisms between OIH and chronic migraine occur through neuropeptide dysregulation. Using label-free, non-biased liquid chromatography-mass spectrometry to identify and measure changes in more than 1500 neuropeptides under these two conditions, we observed only 16 neuropeptides that were altered between the two conditions. The known pro-migraine molecule, calcitonin-gene related peptide, was among seven peptides associated with chronic migraine, with several pain-processing neuropeptides among the nine other peptides affected in OIH. Further, composite peptide complements Pituitary adenylate cyclase-activating polypeptide (PACAP), Vasoactive intestinal peptide (VIP) and Secretogranin (SCG) showed significant changes in both chronic migraine and OIH. In a follow-up pharmacological study, we confirmed the role of PACAP in models of these two disorders, validating the effectiveness of our peptidomic approach, and identifying PACAP as a mechanistic link between chronic migraine and OIH. Data are available via ProteomeXchange with identifier PXD013362.

## Teaser

The neuropeptides within seven nervous system regions involved in relaying pain signals have been identified and quantified in mouse models of chronic migraine and opioid-induced hyperalgesia using label-free peptidomics. Of the >1500 peptides identified, only a few neuropeptides were significantly altered between the two conditions, including the known pro-migraine molecule, calcitonin-gene related peptide. As pituitary adenylate cyclase activating enzyme (PACAP)-related peptides were similarly altered in both groups, we validated PACAP as an overlapping mechanism of chronic migraine-associated pain and opioid-induced hyperalgesia.

## Graphical Abstract

**Figure:**
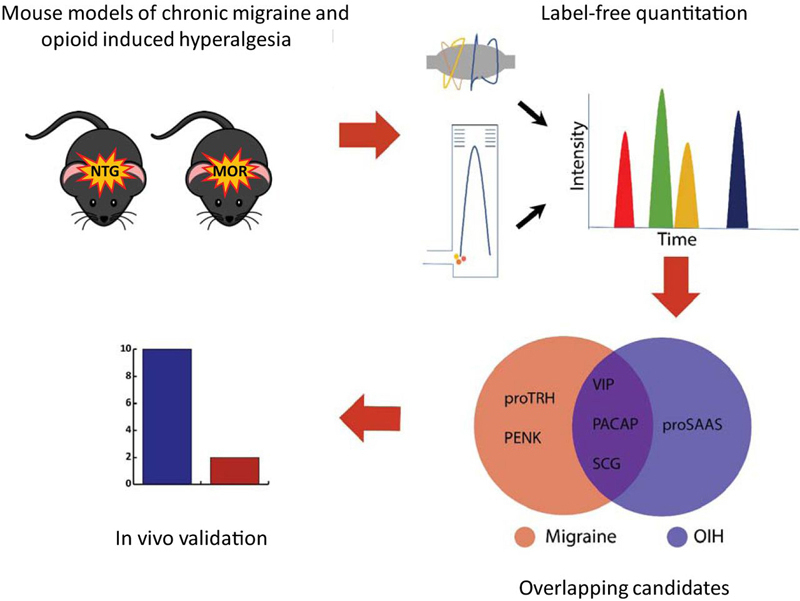

**Highlights**

• Characterized the peptides involved in migraine and opioid-induced hyperalgesia (OIH).
• Identified more than 1500 peptides in seven nervous system regions.
• Discovered seven peptides significantly changed with migraine and nine with OIH.
• Validated PACAP involvement in both conditions via pharmacological studies.

Migraine is among the top twenty most debilitating disorders in the world and is estimated to affect hundreds of million people annually (1). Despite such high prevalence and the concomitant societal burden of several billions of dollars annually in lost productivity and medical costs, the treatments available for this disorder are ineffective for a large proportion of patients (2). In a significant number of cases, opioid-based therapeutics, such as morphine, are prescribed to alleviate migraine (3, 4). Although opioids may provide acute relief, regular use results in addiction/dependence and can lead to refractory headache and contributes to the progression of migraine from an episodic to a chronic condition on extended usage (3, 4). In the field of pain management, opioid-induced hyperalgesia (OIH)^1^ has also been observed, where repeated use of opioids leads to several adverse effects, including an increased sensitivity and expanded area of pain (5). Though opioid withdrawal is a potential solution to combat OIH, previous studies have shown an approximate 50% relapse rate among the patients subjected to opioid withdrawal. The lack of proper mechanistic understanding of OIH and the chronification of migraine as a result of repeated opioid usage is a major roadblock to the development of effective therapies for these conditions. Our goal is to use animal models of these disorders to determine if alterations in neuropeptide dynamics within key regions that regulate nociceptive and emotional processing could provide insight into the underlying mechanisms governing these types of pain (Fig. 1*A*). This knowledge will be useful in developing novel treatment procedures with reduced tendencies to cause dependence/addiction than the existing opioid-based treatments.

**Fig. 1.**
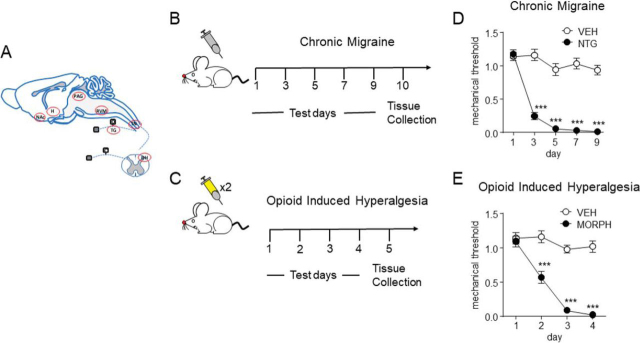
*A*, Nervous system regions collected for the peptide quantitation analysis. DH, dorsal horn; H, hypothalamus; NAc, nucleus accumbens; PAG, periaqueductal gray; RVM, rostroventral medulla; TG, trigeminal ganglia; TN, trigeminal nucleus caudalis. Treatment paradigm for inducing (*B*) migraine-associated pain and (*C*) OIH. Chronic NTG and chronic morphine both produce hyperalgesia. Responses to mechanical von Frey hair stimulation was determined before drug/vehicle injection. *D*, Mice treated every other day with NTG (10 mg/kg, IP). *E*, Mice treated with morphine twice daily (days 1–3, 20 mg/kg, day 4, 40 mg/kg, SC). Both data sets were analyzed by two-way repeated measures ANOVA, *p* < 0.001 time, drug, and interaction, ***p < 0.001 as compared with vehicle day 1 responses. *n* = 15/group.

The leading hypothesis on migraine pathogenesis is based on neuropeptide-mediated modulation of the trigeminovascular and other migraine-related nervous system structures. Compelling evidence from previous studies on gene, transcript or receptor expression points toward the direct involvement of neuropeptides in the regulation of nociception, allodynia and chronic headaches (6, 7). However, a comprehensive evaluation of the brain peptide repertoire, with the goal of understanding their roles in these conditions, has not been performed to date. Peptidomics, a direct global analysis of endogenously present peptides, is particularly illuminating as the approach can be used to evaluate final gene products where they act and thus, provides information that most other 'omics technologies cannot. One of the primary reasons for the slow exploration of neuropeptide dynamics in migraine is that direct label-free approaches for the measurement of peptides, such as mass spectrometry (MS)-based peptidomics, lack the intrinsic signal amplification steps of molecular techniques, and accordingly, require more biological tissue per sample (8). In addition, nervous system regions involved in migraine are numerous, relatively small and hard to isolate reproducibly. Superseding these systematic limitations, we have measured the set of endogenous peptides present in detectable amounts in the following regions–trigeminal nucleus caudalis (TN), nucleus accumbens (NAc), dorsal horn of the lumbar spinal cord (DH), rostroventral medulla (RVM), trigeminal ganglia (TG), hypothalamus (H), and periaqueductal gray (PAG)–and identified small, overlapping, but not identical, subsets of neuropeptides defining migraine or OIH, in both models. For our migraine model, we used chronic intermittent doses of the known human migraine trigger, nitroglycerin (NTG). We (9, 10), and others (11, 12), have previously shown that this model evokes a severe basal hypersensitivity that is responsive to migraine preventives (9, 10). For our OIH model, we administered morphine twice daily for 4 days, with an escalated dose on day 4, a protocol that produces robust OIH (13). The wealth of information obtained with behavioral assessment was combined with MS-powered quantitative peptidomics analysis of global neuropeptide changes and characterized alterations of endogenous peptides derived from several prohormones, such as Pro-calcitonin gene related peptide (Pro-CGRP), Pro-Neuropeptide Y (Pro-NPY), Pro-Enkephalin (PENK), Pro-Opiomelanocortin (POMC) and Pro-Tachykinin (Pro-TKN), thus laying a foundation for future functional studies and, hopefully, treatment. The protein pituitary adenylate cyclase-activating polypeptide (PACAP)-derived peptide was found to be significantly altered in both the migraine and OIH models, further cementing its role in the regulation of pain symptoms. Our results confirm the hypothesis that a shared mechanism, based on neuropeptide dysregulation, exists between these disorders.

## MATERIALS AND METHODS

The study was performed twice, in two independent cohorts separated by one year (cohort 1 and cohort 2). Both these cohorts tested the OIH (morphine) and migraine (NTG) models with identical treatment groups, sample collection protocols, and analysis methods. This approach was used to test robustness of the approach. Only significantly changed peptides and proteins that fell below the *p* value threshold (*p* ≤ 0.05 for peptides and *p* ≤ 0.1 for proteins) in both cohorts were truly correlated to treatment. A third cohort of animals was used to validate one of the overlapping peptides identified in our screen, PACAP, in the chronic NTG and OIH mouse models.

### Animal Treatment

Subjects were male C57BL6/J mice (Jackson Laboratories, Bar Harbor, ME), between 9–12 weeks old. Animals were group-housed on a 12–12 light-dark cycle, and food made available *ad libitum*. Experimental procedures were approved by the University of Illinois at Chicago Office of Animal Care and Institutional Biosafety Committee, in accordance with AALAC guidelines and the Animal Care Policies of the University of Illinois at Chicago, as well as with the European Union directive on the subject of animal rights. Animals were weighed daily during treatment, and no adverse effects of treatment were observed on body weight or on visual examination.

For behavioral experiments, animals were counterbalanced into groups following the first basal test for mechanical sensitivity. The experimenter was blinded to the drug condition being tested. Animal testing occurred in low-light conditions, between 8–15 h. To determine mechanical responses, the threshold for response to punctate mechanical stimuli was tested according to the up-and-down method. Animals were habituated to the testing boxes for 2 days before testing. For hindpaw sensitivity, the plantar surface of the animal hindpaw was tested. For cephalic testing, the periorbital region caudal to the eyes and near the midline was tested. In both cases, the testing region was stimulated with a series of eight von Frey filaments (bending force ranging from 0.008 to 2 g). Groups were counterbalanced based on their naïve baselines (basal responses taken on day 1).

Injections were administered as a 10 ml/kg volume. Other drugs were dissolved in 0.9% saline solution, which was used as the corresponding vehicle control. For chronic migraine, mice were injected with NTG (10 mg/kg, American Reagents, Shirley, NY) or vehicle every other day for 9 days (5 testing days). On test days, baseline mechanical responses were taken before drug administration and post-treatment responses were assessed 2 h post-drug treatment. For OIH, mice received SC injections of morphine or vehicle twice daily for 4 days. On days 1–3 the dose of morphine was 20 mg/kg, and on day 4 it was 40 mg/kg. Baselines were determined before the morning injection of morphine/vehicle.

For PACAP experiments, the PAC1 receptor antagonist, M65 (0.1 mg/kg, Bachem Torrance, CA) or vehicle was administered IP 30 min before testing. To test the effect of M65 in chronic migraine, mice were treated as described above with vehicle or NTG every other day for 9 days. One hour and 30 min post-NTG/VEH they were injected with vehicle or M65. Cephalic allodynia was determined before NTG/VEH administration (basal responses) and 30 min after M65/VEH injections (post-treatment responses) on days 1, 5 and 9. To determine the effect of PAC1 inhibition in the OIH model, mice were treated twice daily with morphine as described above, and cephalic responses were determined before morphine/VEH administration on days 1 and 3. On day 5, 15–18 h after the final morphine/vehicle injection, basal cephalic responses were determined, and 3 h later mice were injected with M65/vehicle and tested 30 min later.

### Tissue Collection

For both models, tissue was collected 24 h following the final injection of drug or vehicle. Mice were anesthetized by injection of Somnasol and then perfused transcardially with ice-cold physiological saline to remove the blood and preserve integrity of live neural tissue (14). Brains were immediately removed and sliced on a cold brain matrix. Brain regions of interest were isolated from brain slices according to Paxinos stereotaxic coordinates using a biopsy punch of a suitable diameter (15); isolated samples were thermally stabilized using a Stabilizor T1 tissue stabilization device (Denator AB, Uppsala, Sweden), transferred into microcentrifuge tubes and frozen on dry ice for the duration of sampling. Samples were stored at −80 °C until use.

### Peptide Extraction

Samples were thawed and homogenized on ice. Before peptide extraction, tissues from 5 animals (cohort 1) and 3 animals (cohort 2) were pooled together to form one biological replicate. These pooled samples were then subjected to a three-stage peptide extraction (16). The peptide extracts were desalted using C18 spin columns (Thermo Fisher Scientific, Waltham, MA). Following the desalting, the samples were vacuum dried and stored at −20 °C until further use.

### Mass Spectrometric Measurement Design

Two nanoLC-nanoESI-MS platforms were used in the study: an Orbitrap Fusion Tribrid (Thermo Fisher Scientific) was used for peptide sequencing/identification and library construction from pooled aliquots of all individual samples (10%v/v), and an IMPACT HD Q-TOF (Bruker, Billerica, MA) for quantitation of peptides in individual biological replicates by peak area of precursor ions in MS1 scans. Peptide libraries built from the first cohort samples were used as a reference to quantify the corresponding peak areas in MS1 spectra acquired from both first and second cohorts, as described in the next section. Technical justification of our experimental design is that Orbitrap mass analyzers are known to be influenced by space charge effects which could potentially affect the quantitation results. QTOFs that do not trap ions are not prone to space charging and offer advantage in quantitative application. It has also been shown that Orbitrap mass analyzers have a reduced intrascan dynamic range compared with TOFs (17). Besides, analysis of MS1 scans is straight forward and robust, which was critically important with the number of samples to measure and quantify (7 regions × (3 biological replicates cohort one + 5 biological replicates cohort 2) × 4 conditions = 224).

### LC-MS and LC-MS/MS Analysis

Each of the vacuum dried samples was reconstituted in 10 μl of water supplemented with 0.1%(v/v) formic acid and subjected to LC-MS analysis. Briefly, for identification, the samples were separated on a Dionex Ultimate 3000 nanoLC system (Thermo Fisher Scientific) using a reversed phase C18 column (PepMap Thermo Fisher Scientific 75 μm × 15 cm, C18, 2 μm, 100 Å) with a solvent gradient consisting of solvent A (H_2_O with 0.1% formic acid (FA)) and solvent B (acetonitrile with 0.1% FA) at 300 nl/min. The runs lasted for 120 min with the following gradients: 0–3 min, 1–1% B; 3–6 min, 1–10% B; 6–90 min, 10–70% B; 90–100 min, 70–99% B; 100–110 min, 99–1% B; 110–120 min, 1–1% B. Data was acquired using an nanoESI (electrospray ionization) source in a data-dependent MS/MS mode via collision-induced dissociation (CID) over an *m/z* range of 200–1200 with dynamic exclusion turned on (exclusion after 2 spectra within 30 s.). However, the ion is reconsidered if it elutes after 60 s. or later. The parent ions were scanned with an Orbitrap resolution of 120K with an AGC target value of 200,000. Precursor ions with a charge ranging from +1 to +7 were considered. A normalized collision energy value of 35% was used for fragmentation via collision-induced dissociation. For the quantitation run, the same LC system was hyphenated to a Q-TOF mass spectrometer via a CaptiveSpray source (Bruker), and the separation method was exactly the same as used for the identification experiments on the Orbitrap instrument. The data were acquired in MS (MS^1^) mode over a range of 290–3000 *m/z* with a cycle time of 3 s. A fixed MS^1^ scan rate of 1 Hz was used. An absolute intensity threshold of 694 counts (100 per 1000 sum) was used for spectra collection.

### Peptide Identification and Tissue-specific Peptide Libraries

The acquired .raw files from the Thermo Orbitrap Fusion were directly imported (without any peak filtering) into PEAKS 8.0 software (Bioinformatics Solutions Inc., Waterloo, ON, Canada) and searched against a mouse proteome database with 81,515 proteins that was downloaded from UniProt (18) for identification. The following database search parameters were used: precursor mass tolerance, 20 ppm; fragment mass tolerance, 0.1 Da; no enzymatic cleavage; variable post-translational modifications (PTMs), including acetylation (K and N-terminal), amidation, phosphorylation (STY), half-disulfide bond (C), pyroglutamination (E and Q)and Met oxidation (M); maximum number of variable PTMs, 3. A FDR threshold of 1% (for peptide-spectrum matches (PSMs)) was used to filter the identified peptide sequences. The FDR was estimated by the PEAKS target-decoy approach. The list was then manually validated to remove possible false positives for quantitation. These include incorrect PTMs, such as C-terminal amidation, for peptides where the subsequent amino acid is not a glycine, caused by the statistical nature of sequence assignment by the search engines (19). A total of over 200 LC-MS runs were performed on seven different regions in two independent cohorts.

### Calculating Peptide Peak Areas Via Skyline

The peptide peak areas were calculated using Skyline software (20). A Skyline library was built for each region of interest, providing information on identified peptides, including their *m/z*. After peptide library construction, LC-MS data files related to the different studied regions and conditions were imported into their corresponding Skyline projects. For peptides present in the library, corresponding extracted ion chromatograms (XIC) were generated from every single LC-MS file by Skyline. The integrated peak areas of XIC were used for peptide quantitation. The peak areas of signals corresponding to different charge states of the same peptide were summed. The summed peak areas were log2 transformed and exported to Microsoft Excel for further statistical analysis. This transformation was performed to ensure that the data follows a normal distribution (which is a required condition for several downstream statistical methods). Correlation between the two independent cohorts was evaluated by comparing the mean peak area values corresponding to peptide signals acquired from both the cohorts via Pearson correlation factor using MATLAB.

### Statistical Rationale for Peptide Quantitation and Behavioral Analysis

Peptide quantitation data exported from Skyline (*n* = 3 for cohort 1 and *n* = 5 for cohort 2) was normalized using a locally weighted regression analysis. The second cohort was intended to validate findings from the first cohort, and the data were analyzed within and between cohorts. Normalization was performed using the online tool Normalyzer (21). This tool performs a locally weighted regression (LOESS) to account for systematic bias in the dataset. An unpaired, 2-tailed Student's *t* test with equal variance was used to identify the significantly different peptide levels in the control and test groups for both conditions (supplemental Table S2 and on ProteomeXchange Consortium via the PRIDE). The peptides that fell below the set value (*p* ≤ 0.05) in both independent cohorts were identified to be significant (supplemental Table S3). Peptides that had *p* ≤ 0.05 in both cohorts should have a probability that this occurs because of random chance of <0.05*0.05 = 0.0025, and so further multiple testing correction was not performed. The composite prohormone profile differences between the treatment and control groups for both cohorts were evaluated by summing the peak areas of the detected endogenously cleaved peptides from a given precursor protein and comparing the summed areas. An unpaired, 2-tailed Student's *t* test was performed on the resultant total precursor protein values and a cutoff of *p* ≤ 0.1 was used to find proteins with significantly different levels (supplemental Table S3). Even in this case, only precursor proteins that had *p* ≤ 0.1 in each of the two cohorts (or ≤.01 overall probability) were considered. Additional Supplementary tables (S5 and S6) are also included that depict the protein coverage (5) and group of proteins that are mapped to the same peptide sequence (6). For the animal experiments, a 2-way repeated measures ANOVA was used to analyze chronic effects of treatment. Acute effects of M65 following OIH were evaluated using 2-way ANOVA. Analyses were performed in SigmaStat.

## RESULTS

### Behavioral Model Validation

**Fig. 2.**
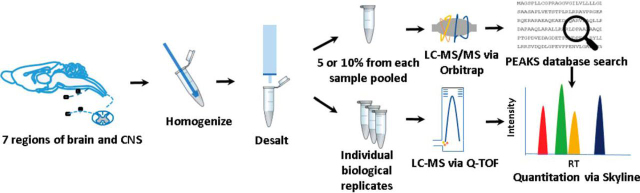
**Experimental design and workflow for tissue collection and peptide extraction followed by label-free MS-based quantitation.** Regions of the nervous system were processed and analyzed individually.

The study was performed twice on two independent animal cohorts treated and assayed one year apart (*N_total_* = 120 animals; cohort 1: 3 biological replicates × 4 conditions × 5 animals per replicate = 60; cohort 2: 5 biological replicates × 4 conditions × 3 animals per replicate = 60). The second cohort was intended to validate findings from the first cohort, and the data were analyzed within and between cohorts. To induce chronic migraine-associated pain, animals were given an intraperitoneal (IP) injection of the known human migraine trigger, NTG (10 mg/kg) (migraine), or vehicle (migraine-VEH) every other day for 9 days (5 administrations total) (Fig. 1*B*). On the treatment days, mice were tested before NTG administration; NTG, but not migraine-VEH, produced a significant basal mechanical hyperalgesia (Fig. 1*D*). For OIH, mice were treated twice daily with morphine or vehicle (OIH-VEH). On days 1–3 they received 20 mg/kg morphine via subcutaneous (SC) injection, and on day 4, 40 mg/kg morphine (Fig. 1*C*). Mechanical responses were determined before the morning injection; morphine treatment also produced a significant mechanical hyperalgesia (Fig. 1*E*). Targeted nervous system regions (Fig. 1*A*) were collected from both cohorts 18–24 h after the final drug/control treatment, and processed as shown in Fig. 2.

### Pain-related Peptide Complement as Detected by Liquid Chromatography (LC)-Tandem Mass Spectrometry (MS/MS)

**Fig. 3.**
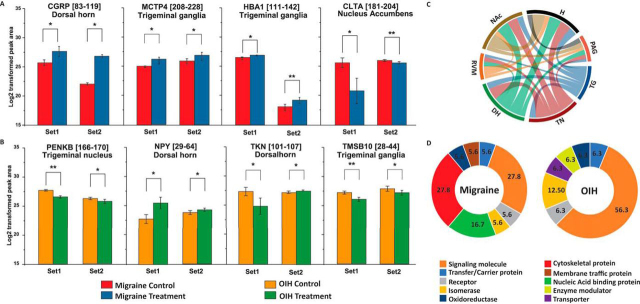
**Select peptide level changes as determined by quantitative mass spectrometry for (*A*) migraine and (*B*) OIH models.** *p ≤ 0.05, **p ≤ 0.01. The histogram values represent the Log2 transformed value of each peptide signal. Error bars represent standard deviation of mass spectrometric signals corresponding to the respective peptides. Full list of altered peptides given in supplemental Table S3. *C*, Chord diagram of neuropeptide overlap detected among regions of the nervous system: H: hypothalamus; PAG: periaqueductal gray; TG: trigeminal ganglia; TN: trigeminal nucleus; DH: dorsal horn; RVM: rostroventral medulla; NAc: nucleus accumbens. In this figure, the thickness of the lines between two regions is directly proportional to the extent of overlap between two regions. *D*, Functional classification of select proteins affected by migraine and OIH conditions. Protein selection was based on detection of significant level differences among protein-derived peptides detected in samples of the nervous system regions collected from migraine and OIH animal models.

Endogenous peptide *de novo* sequencing facilitated by LC-MS/MS and followed by bioinformatic data interpretation led to the structural characterization of >1500 chemically unique peptides in morphologically defined regions of the central and peripheral nervous systems that are associated with nociceptive and emotional pain processing (supplemental Tables S1 and S2) as follows: TN (605), NAc (550), DH (528), RVM (150), TG (1376), H (967) and PAG (156). A high degree of overlap between detected peptides in region-specific subsets was observed (Fig. 3*C*). This data represents the most extensive cataloguing to date of endogenous peptides relevant to migraine and OIH. Pain-related regions of the nervous system previously investigated for their native peptide complement under different biological paradigms are the DH, H, and NAc, whereas the peptidome of other regions tested here have not been previously described in the literature. Comparing against the neuropeptide gene expression data found in the Allen Brain Atlas (22), these results validate the presence of final gene products in the DH, H, NAc, and PAG regions, and provide new insights on neuropeptides in the TG and TN region that have not been profiled previously.

### Quantitation of Neuropeptide Dynamics

Following peptide identification, quantitation of peptide levels was performed using Skyline software (20). Skyline spectrum libraries were built for each assessed brain region based on MS/MS data from the peptide identification experiment described above. The two animal cohorts described in the behavioral section were quantified separately; results from the initial discovery cohort were independently validated by the results obtained from the second cohort. The tissue-specific data sets demonstrated a strong positive correlation between treatment groups and animal cohorts (Pearson's correlation factor (ρ) between 0.5–0.9), with the exceptions of the TG (ρ = 0) and NAc (ρ = 0.3–0.4) (correlation maps are provided in the Supplementary Information). Among the peptides with quantifiable signals and detected in at least half of the biological replicates (1356 peptides for the migraine group and 1397 peptides in the OIH group) from the samples collected in both the cohorts, 16 were significantly different (*p* ≤ 0.05, supplemental Table S3) between the migraine/migraine-VEH and OIH/OIH-VEH groups. Compared with the migraine-VEH group, NTG-treated animals showed a significant increase or decrease in the following peptides in different regions (with full name and UniProt accession numbers in parenthesis): DH - CGRP ([83–119]) (Calcitonin gene related peptide1 Q99JA0) and SCGI [356–374] (Secretogranin-1 P16014), PAG - PENK A[197–208] (Pro-Enkephalin A- P22005), TG - HBA1[111–142] (Hemoglobin Subunit alpha1 Q91VB8) and MCTP4[208–228] (Mast cell protease-4 P21812), NAc - CLTA[181–204] (Clathrin light chain A- O08585) and TUBB5 [137–167] (Tubulin beta-5 P99024) (Fig. 3*A*). Similarly, animals with symptoms of OIH showed significant increases or decreases in the following peptides in various tested regions: DH - NPY[29–64] (Pro-Neuropeptide Y P57774), TKN[101–107] (Pro-Tachykinin P41539) and SCGI [333–355] (Secretogranin1 P16014), TG -TMSB10[28–44] (Thymosin-β 10 Q6ZWY8), PAG - proSAAS [45–59] (Q9QXV0) and SCG1[437–454](P16014), TN - SCG [333–355] (P16014) and PENKB [166–170] (Pro-EnkephalinB/Pro-Dynorphin-O35417), PAG - and NAc - AN32A [1–11] (Acidic nuclear phosphoprotein pp32-O35381) (Fig. 3*B*). Because only the peptides with a *p* value ≤0.05 in both cohorts were chosen, a further false discovery rate (FDR) correction was not applied. In summary, significant differences in peptide levels were found in the DH, NAc, TG, and PAG regions for the migraine model, and in the DH, NAc, TG, PAG, and TN regions for the OIH model.

HBA1 [111–142] (Q91VB8), identified here, is a novel peptide candidate that has not been reported in any of the previous pain-related studies and could potentially be involved mechanistically in chronic pain disorders (23) because of its similarity with neokyotorphin peptides. Also, in the current study, we observed a significant change in the level of a 7-residue-long peptide, (TKN[101–107])(P41539), in the DH region of the migraine model, compared with the control. This C-terminal fragment of the longer Neurokinin-A peptide is also a novel candidate and shares the same C-terminal sequence (Phe-X-Gly-Leu-Met-NH_2_) with other known peptide agonists of the tachykinin receptor.

Interestingly, seven of the peptides (Migraine: DH-SCGI [356–374](P16014) and PAG - PENK A[197–208](P22005); OIH: NAc - AN32A [1–11](O35381); PAG - proSAAS [45–59](Q9QXV0), SCG[437–454](P16014); DH- TKN[101–107] (P41539) and TN-SCG [333–355](P16014)) with significantly altered levels between migraine/migraine control and OIH/OIH control groups showed differences in the opposite direction between the two cohorts measured. Because we detected more than 1500 peptides, and only a small subset of the same peptides was found to be significantly different among tested conditions, it is likely that these peptides are significant, but the specific levels could be impacted by biological rhythms, seasonal changes or other variables.

### Functional Classification of Migraine and OIH-relevant Precursor Proteins

The precursor proteins (not just prohormones) for the significantly changed peptides in cohort 1 and cohort 2 were considered for functional classification. From this list of proteins, the ones that showed up consistently in both cohorts were then subjected to gene ontology (GO) classification. This resulted in 31 (24 if the same protein from different regions is counted only once) precursor proteins for migraine and 24 (18 if the same protein from different regions is counted only once) for OIH (corresponding to peptides with *p* ≤ 0.05, Table I). These did not match with the peptide numbers for two reasons. (1) If a different peptide from the same precursor protein was significantly changed in cohort 1 and cohort 2, we did not consider the peptide to be a hit because it was not consistent in both the cohorts; however, the precursor protein was taken into account for GO classification. (2) There are several peptides that map to multiple distinct proteins and we cannot know which one they belong to; thus, each of them was considered separately. These 31 and 24 precursor proteins corresponding to migraine and OIH conditions, respectively, were evaluated for GO using PANTHER analysis (24). We found that migraine primarily induced changes in cytoskeletal proteins, signaling molecules, nucleic acid binding, oxidoreductase, receptor, transfer/carrier protein, isomerase and membrane traffic proteins. OIH also disproportionally affected signaling proteins, as well as receptor, transporter, oxidoreductase, transfer/carrier protein, isomerase and enzyme modulating classes of proteins. Intriguingly, six precursor proteins (determined by the overlap between protein classes among treatment groups from PANTHER analysis) were common to both migraine and OIH treatments in both cohorts, and include SCG, PENK, proCGRP, VGF, and Peptidyl-prolyl-cis-trans-isomerase-A.

**Table I:** Precursor proteins that correspond to the significantly changed peptides for the migraine and OIH models. For this analysis, the list of precursor proteins that are common to both cohort 1 and cohort 2 only were considered. Gene expression validation (from Allen Brain Atlas) of the same proteins is included in Supplementary Table IV

Region	Protein name (Migraine)	Protein name (OIH)
DH	Secretogranin I	Secretogranin II
	Pro-Enkephalin A/B	Secretogranin I
	Calcitonin gene-related peptide	Pro-Enkephalin A/B
	Macrophage migration inhibitory factor	Calcitonin gene-related peptide
	Phosphatidylethanolamine-binding protein	Pro-Neuropeptide Y
		Protachykinin-1
H	Secretogranin I	Secretogranin II
	Eukaryotic translation initiation factor	Neuro VGF
		Neuroendocrine protein 7B2
		ProSAAS
NAC	Macrophage migration inhibitory factor	Peptidyl-prolyl cis-trans isomerase A^a^
	Phosphatidylethanolamine-binding protein	Neuro VGF
	Clathrin light chain^a^	Acidic leucine-rich nuclear phosphoprotein 32 family member A
	Serine/arginine-rich splicing factor	Vasoactive intestinal peptides
	Tubulin beta-2A chain^a^	Peptidyl-prolyl cis-trans isomerase^a^
	Tubulin beta-2B chain a	
	Tubulin beta-6 chain	
	Tubulin beta-5 chain^a^	
	Tubulin beta-4B chain^a^	
	Clathrin light chain^a^	
	Clathrin light chain^a^	
	Calcitonin gene-related peptide^a^	
	Clathrin light chain^a^	
	Clathrin light chain^a^	
PAG	Pituitary adenylate cyclase-activating polypeptide	ProSAAS
	Pro-Enkephalin A/B	Secretogranin I
	Neuro VGF	
	Secretogranin II	
TG	40S ribosomal protein S12^a^	Thymosin beta-10
	Mast cell protease 4	Apolipoprotein A
	Alpha Globin	
	Peptidyl-prolyl cis-trans isomerase A	
	Hemoglobin A^a^	
TN	Calcitonin gene-related peptide	Pro-Enkephalin A/B
		Neuro VGF
		Secretogranin I
		ProSAAS
		Pro-Dynorphin

a Precursor proteins where the peptide is mapped to more than one protein.

### Neuropeptide Prohormone Composite of the Migraine and OIH Conditions

**Fig. 4.**
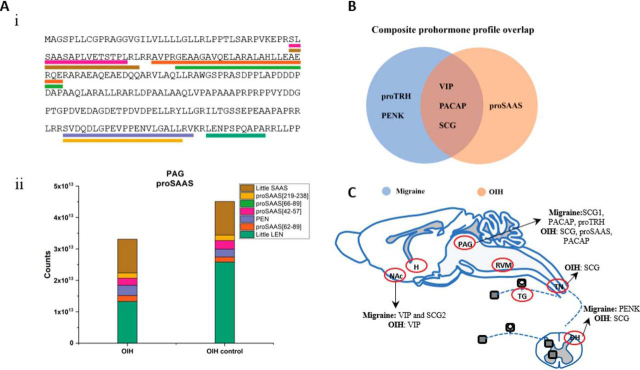
**Composite prohormone profiling.***A*, An example of proSAAS prohormone with the detected, quantified peptides originated from conventional mono/dibasic cleavage sites (i); quantification of the prohormone from additive signal of detected constituent peptides of proSAAS for the OIH model in the PAG (ii). *B*, Overlap of the prohormones with the composite prohormone signature significantly different (*p* ≤ 0.1) between the treatment and control groups for both migraine and OIH. VIP, SCG and PACAP are common to both migraine and OIH conditions. proTRH and PENK are exclusive to the migraine group whereas proSAAS is exclusive to the OIH group. *C*, Studied regions labeled with the prohormones that are significantly different between the control and treatment groups.

Prohormone processing often creates multiple biologically active peptides; whereas as expected peptides from a prohormone are cleaved, they can be differentially packaged and transported, released at different sites, and thus, have different activities and lifetimes. Considering that the additive effects of subtle changes in the levels of individual peptides may lead to significant cumulative effects in the system, we quantified the precursor protein peptide composites (cumulative changes in detected peptides from a prohormone) for both cohorts. For this profiling effort, the sum of detected peptide signals (peak areas corresponding to peptide ions as measured by a mass spectrometer) corresponding to mature, endogenously cleaved (based on known prohormone processing rules) and detected peptides from a given prohormone were compared between the migraine/migraine-VEH and OIH/OIH-VEH groups (Fig. 4*A*). Using this approach, a total of 6 prohormones were found to correlate with migraine and/or OIH conditions from their respective controls: Vasoactive Intestinal peptide (VIP), PACAP, thyrotropin-releasing hormone prohormone (proTRH), PENK, SCG and proSAAS (Fig. 4*C*). Specifically, for migraine: VIP and SCG2 in the NAc, PACAP, SCG1, and proTRH in the PAG, and PENK in the DH; and for OIH: VIP in the NAc, SCG, proSAAS and PACAP in the PAG, and SCG in the TN and DH, were significantly changed between the treated and control groups (*p* ≤ 0.1) in both cohorts (supplemental Table S3 and Fig. 4*B*). The only individual peptide that was from one of the above-mentioned prohormones and significantly altered by itself was PENK A[197–208](P22005) in the PAG for the migraine model. In all other prohormones, the individual peptides constituting the prohormone did not show significant changes whereas the composite profiling was significantly different. The current discovery could provide more insights about the role of the NAc in pain processing. Although PACAP was found in several nervous system regions, significant differences in its derived peptide levels were only observed in the PAG in both the migraine and OIH models. Prohormone level changes correlated to migraine were observed for proTRH-releasing hormone exclusively in the PAG, although proTRH-derived peptides were also detected in examined tissues, except the TG. Another migraine-exclusive change in composite prohormone level involves PENK in the DH. In contrast, the OIH condition was characterized by composite proSAAS peptide level changes selectively in the PAG (supplemental Table S3).

### Validation of Detected Neuropeptide Localization in the Nervous System

To verify that differentially detected prohormones in the migraine and OIH models are present in the analyzed regions of the nervous system, we checked the gene expression profiles of the DH, H, NAc, and PAG found in the Allen Brain Atlas (22), as well as in the literature. This comparative analysis indicates that genes or transcripts corresponding to the most detected peptides in this study are indeed expressed in the DH, H, NAc, PAG, and TG (supplemental Table S4). However, a few of them, such as Macrophage migration inhibitory factor (MIF), AN32A and VIP in the NAc are not expressed in that region according to the Allen Brain Atlas. Thus, our MS findings revealed that these peptides might have been transported to the NAc from a different region of the nervous system.

### In Vivo Validation of Overlapping Pain Mechanisms for Migraine and OIH

**Fig. 5.**
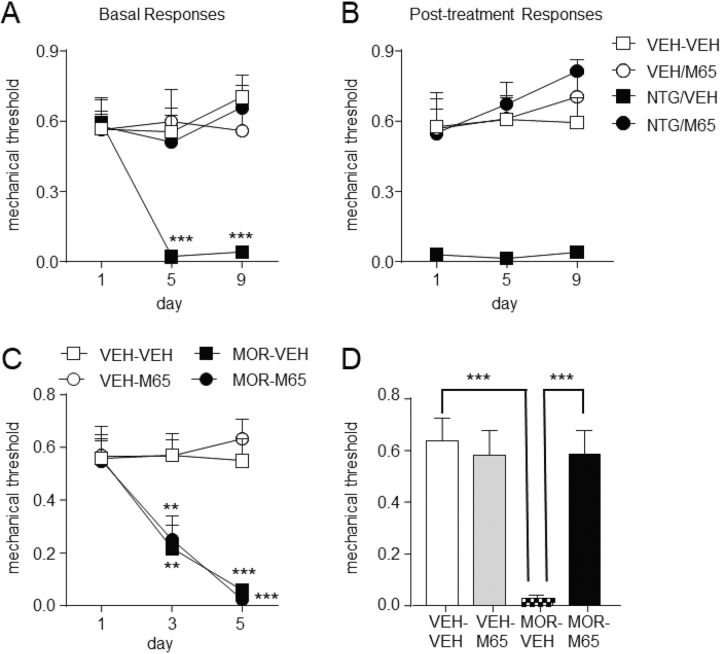
**PAC1 inhibition by M65 blocked pain induced by chronic NTG and morphine.** To induce chronic migraine-associated pain, mice were treated every other day, but periorbital allodynia was determined only on days 1, 5, and 9. Allodynia was assessed before treatment (*A*, basal responses) and 2 h after drug administration (*B*, post-treatment responses). M65 (0.1 mg/kg IP) or vehicle (VEH) was injected 1 h, 30 min post-NTG/VEH. (A) M65 blocked the development of basal hypersensitivity, 2-way repeated measures ANOVA, *p* < 0.01 treatment and interaction, ***p < 0.001 as compared with VEH-VEH on day 1; and (*B*) acute NTG-induced allodynia, 2-way repeated measures ANOVA, *p* < 0.001 treatment only. *C*, To evoke OIH, mice were administered morphine or vehicle twice daily for 4 days (days 1–3, 20 mg/kg; day 4, 40 mg/kg, SC), but periorbital allodynia was only assessed on days 1 and 3 of treatment, and 15–18 h after the final treatment (day 5). Morphine treatment caused significant periorbital allodynia, 2-way repeated measures ANOVA, *p* < 0.01 drug, time and interaction, **p < 0.01, ***p < 0.001 as compared with VEH-VEH on day 1. *D*, Administration of M65 24 h after the last injection of morphine/VEH (day 5) reversed OIH, 2-way ANOVA, *p* < 0.01 pretreatment (VEH/morphine), drug (M65/VEH), and interaction, ***p < 0.001. *n* = 6/group. PACAP is an overlapping mechanism involved in the development of both chronic migraine-associated pain and opioid-induced hyperalgesia.

To validate the hypothesis that there are overlapping mechanisms between chronic migraine and OIH, we examined the effect of PACAP, one of the prohormones with a peptide composite profile that was similarly altered in both the migraine and OIH groups (Fig. 4*B* and 4*C*), within our behavioral models. PACAP can bind to three different receptors; however, because PAC1 is the receptor most commonly associated with migraine-related effects (25, 26), we tested the PAC1 inhibitor M65 in our chronic migraine and OIH models. Mice were tested for cephalic (periorbital) mechanical responses before (basal) and 2 h post-NTG treatment. M65 (0.1 mg/kg, IP) was administered 1 h and 30 min post-NTG/VEH injection. Animals were treated every other day for 9 days, but only tested on days 1, 5, and 9. Chronic intermittent NTG treatment induced periorbital allodynia basally (Fig. 5*A*) and 2 h post-treatment (acute responses, Fig. 5*B*); these effects were completely blocked by M65. For OIH, mice were treated as above for 4 days with morphine or vehicle. Periorbital allodynia was assessed on days 1 and 3 of treatment, and 18 h following the final injection (day 5, Fig. 5*C*). Following the baseline assessment on day 5, mice were injected with vehicle or M65 and assessed 30 min later. Like its effects in the migraine model, M65 completely inhibited OIH (Fig. 5*D*). Our quantitative MS data demonstrate differences in PACAP in samples from both models and controls. The pharmacological and peptidomic measurement outputs together validate our screening approach and point to PACAP as one of the key prohormones involved as an overlapping mechanism of chronic migraine-associated pain and OIH.

## DISCUSSION

Currently available opioid-based therapies are highly addictive, and over-prescription has led to a devastating public health crisis. In the United States, morphine, hydrocodone, and oxycodone are still regularly prescribed for headache (3, 4), and although they may be effective acutely, chronic use results in more frequent and severe headaches that are difficult to treat (3, 4). Medication overuse headache is a form of OIH, and ∼15% of migraine patients develop this disorder (27). The primary treatment for this type of OIH is withdrawal from opioids; however, clinical studies have reported a 20–50% relapse rate within the first year of withdrawal (28, 29), and the majority of patients relapse within the first six months (28). There is clearly a need to understand the mechanisms that underlie the interaction between opioids and migraine in the effort to prevent further drug abuse.

The major goal of the current work was to combine behavioral pharmacology and a large-scale LC-MS approach to look for overlapping mechanisms between chronic migraine and OIH. Our results have provided novel insights on the role of neuropeptides and neuropeptide precursors in migraine and OIH independently, as well as determined the overlapping peptidomic changes between these two disorders. Of the small subset of neuropeptides that we identified in both chronic migraine and OIH, we targeted PACAP for further characterization. We validated this target in both of our models of chronic migraine and OIH, thus bringing the research back to behavioral endpoints. PACAP has been identified as a target for migraine, but has never been considered for OIH, and our work indicates that it may be a bridge linking these two highly prevalent disorders.

Although the epidemiology of migraine has been investigated for several decades (1), the molecular events leading to this neurological disorder are not fully characterized. Some studies have investigated the differences in individual neuropeptides associated with migraine. The best characterized example is CGRP, the levels of which are significantly increased in the venous blood during a migraine attack, as well as in the interictal period in chronic migraine patients (6). Further, neuropeptides from prohormones such as cholecystokinin (30) have also been implicated in the development and maintenance of OIH. However, a limitation of examining the role of individual peptides in a disease state is the need to decide a priori which peptides will be investigated. In addition, most studies use targeted approaches, often relying on indirect measurements using molecular probes such as immunohistochemistry (IHC), which are prone to false positives because of cross-reactivity. Moreover, the antibodies used in IHC studies bind to a relatively small, specific epitope of the peptide of interest and may not be able to discriminate the full-length or post-translationally modified biologically active peptides *versus* their truncated (and potentially inactive) versions.

To overcome these limitations, we used a non-targeted, label-free MS-based approach to profile a large peptide complement of the nervous system regions of interest and measure the change in levels of identifiable peptides. To increase the statistical power and test for the reproducibility of our results, we tested and measured two independent cohorts, revealing a high level of correlation between the cohort results (supplemental Fig. S1) while also validating the newly discovered peptide subsets correlated to migraine and/or OIH conditions. The quantitation results obtained from our experiments provide convincing evidence that differences in the levels of several peptides and precursor proteins correlate with migraine and OIH.

Several previous studies have shown that peptides from prohormones such CGRP (31) (in plasma and TG), NPY (32) (in dorsal root ganglia and DH), and PENK/POMC (33, 34) (in H) are directly associated with migraine and other pain states. We observed significantly higher levels of full-length CGRP peptide (CGRP[83–119])(Q99JA0) in the DH region in the migraine model. Further, in the OIH group, we found a significant decrease in PENK in the TG. YGGFL, a potential biomarker candidate identified in the current study, also known as Leu-enkephalin, is a biologically active peptide derived from PENKB[166–170](O35417) prohormone. Mansour *et al.* (35) have shown that the YGGFL core is necessary and sufficient for a peptide to act as an agonist for both μ- and δ-opioid receptors. A decrease in the endogenous opioid would correspond with increased pain sensitivity. As a corollary to this result, we have previously demonstrated that activation of the δ-opioid receptor can inhibit migraine-associated hyperalgesia in mice (36). In addition, we also observed that Pro-NPY-derived peptide (NPY[29–64])(P57774) was significantly higher in the OIH groups relative to vehicle controls in the spinal cord. NPY has been implicated as an endogenous pain-relieving peptide (32, 37, 38) and shown to be regulated in response to alcohol and drug abuse (39), and its alteration in the OIH model may serve as a compensatory response to the hyperalgesia induced by chronic morphine.

Intriguingly, HBA1[111–142](Q91VB8) was significantly altered between the migraine treatment and control groups. Several studies have demonstrated the bioactive role of hemoglobin-derived peptides. Particularly, the 4–8 residue-long hemorphin and neokyotorphin peptides were shown to produce opioid-like activity (40). HBA1[111–142](Q91VB8), a C-terminal peptide of the hemoglobin subunit-α, shares the terminal 5 residues (Thr-Ser-Lys-Tyr-Arg) with neokyotorphin, which exhibits analgesic properties similar to leu-enkephalin, a well-known endogenous opioid peptide (23).

Peptides of the tachykinin family are well known to be involved in relaying pain signals. Substance P (TKN [58–68])(P41539) is an 11 amino acid-containing peptide that plays a major role in the transmission of nociceptive input (41). Neurokinin-A (TKN[98–107])(P41539) is another peptide from the same precursor protein and is involved in pain perception (42). Both act as an agonist for the tachykinin receptor. The binding of these peptide ligands to the receptor is associated with the transmission of stress, pain, muscle contraction and inflammation signals (41). The known tachykinin receptor peptides possess a common C-terminal structure (Phe-X-Gly-Leu-Met-NH_2_). In the current study, we did find TKN[101–107](P41539), a C-terminal amidated peptide that contains the above mentioned common terminal amino acids.

Other peptides with significantly different levels within our migraine or OIH models were derived from functionally distinct proteins such as SCG, proSAAS, Thymosin-β, Acidic nuclear phosphoprotein, Tubulin-β, Clathrin light chain A, and mast cell protease. Though not directly related to pain mechanisms, the proteins SCG, proSAAS and Thymosin-β are known to produce bioactive peptides that mediate secretory granule formation, wound healing, inflammation and maintenance of the circadian rhythm (43., 44., 45.). The peptides identified from these precursor proteins in the current study could be the novel targets in studies of chronic pain disorder mechanisms. Lastly, some of the precursor proteins such as Tubulin-β, Clathrin light chain A and mast cell protease do not have previous evidence of producing signaling peptides. It is known that peptides transiently exist inside the proteasome of the cell, a multisubunit complex that converts proteins into peptides. It is thought that most proteasome-produced peptides are rapidly degraded in cytosol with a half-life of several seconds, whereas some bind peptide transporters for translocation into endoplasmic reticulum where they bind to major histocompatibility complex I, and are then transported to the cell surface to serve in antigen presentation. At any given snapshot of time when dissection occurred, it is reasonable to expect a repertoire of peptide products of protein catabolism in the sampled tissue. Besides, restructuring of neuronal nets under pathophysiological conditions is not uncommon and would include protein turnover.

Mass spectrometry cannot inform on the origin of detected peptides beyond their association with a protein/s. For peptide quantitation, we standardize sample collection (dissection/tissue transfer time, tissue amount and location by stereotaxic coordinates) and apply the most advanced method of tissue preservation intended to halt postmortem protein degradation by rapid heat deactivation (46). As with any study, there is an assumption here, and it is that postmortem protein degradation, if it occurs, it is at a similar extent in all biological replicates tested. Future studies equipped with this new knowledge on potentially affected peptides may focus on investigation of the involvement of specific proteins in the migraine/OIH paradigm.

As one possibility for the difference in the direction of change for several peptides, there was a significant seasonal difference in the collection of cohort 1 and cohort 2 samples, and this disparity can be in part explained by organism adaptation to slightly different environmental conditions. These seven peptides are truncated versions of full-length mature neuropeptides derived from prohormones such as SCG, Pro-TKN and proSAAS. Three of the seven peptides are from the prohormone SCG, which is known to have unconventional cleavage sites (non-dibasic) (47). The SCGs do not solely serve as peptide precursors and likely have other functions (47). The variable complex cleavage patterns may have resulted in differential changes in levels for the peptides derived from SCG. Regardless of the reasons, the significant changes we observed suggest that these peptide levels are sensitive to the pathophysiological paradigms tested.

Comparing the composite prohormone profile also revealed some unexpected candidates that could serve as bridges between chronic migraine and OIH. The sum of the peak areas of the endogenously cleaved peptides from the prohormone VIP were significantly different between the control and treatment groups of migraine and OIH in the NAc region. VIP and PACAP share homology and are both potent vasodilators (48). However, unlike PACAP, infusion of VIP does not induce migraine (49). VIP has also not been heavily implicated in morphine-related effects, although infusion of VIP into the brain can decrease morphine-induced antinociception (50). Changes in VIP levels in the NAc could indicate that the peptide plays a role in the emotional response to hyperalgesia induced by chronic morphine or NTG and bears further exploration.

PACAP acts as a vasodilator and has a similar physiological role to CGRP in inducing migraine (26, 48, 51). In the current study, we noticed a significant difference between the cumulative peptide signal corresponding to PACAP prohormone in the PAG between the control and treatment groups of both migraine and OIH. This similarity in the change of cumulative peptide signal values for both migraine and OIH groups strengthens the hypothesis that there could be a common underlying mechanism for both disorders. To further elucidate the role of PACAP in chronic migraine and OIH, we tested a PAC1 inhibitor within the studied models. Pharmacological inhibition of the PACAP receptor, PAC1, blocked hyperalgesia induced by either chronic NTG or morphine. Although PACAP has been implicated in migraine pathogenesis, it has not yet been identified as a contributing factor to OIH. Our results indicate that PACAP could be an overlapping mechanism through which opioid treatment can lead to migraine chronification. These results have important translational significance, as the PACAP-PAC1 system is currently being targeted for pharmacological treatment of migraine (26, 48, 51), and our results indicate that these therapies could also be effective in OIH and in medication overuse headache induced by opioid treatment. Moreover, recent studies have also shown that administration of PAC1 receptor agonist at the bed nucleus of stria terminalis can facilitate relapse following extinction of cocaine-seeking behavior (52) which further strengthens our claim on the mechanistic role of PACAP in addiction.

Overall, this study is the first massive exploratory neuropeptide characterization consisting of more than 200 LC-MS experiments resulting in quantitative analysis of around 1500 peptides to assess their association with migraine and OIH. Our results confirm and expand our understanding of the involvement of known and suspected molecular players in migraine and OIH and add important details on other peptides. Our data identify lead peptides and prohormones that mechanistically bridge the gap between opioid use and migraine chronification and serve as a solid foundation for future studies related to pain processing. Our study also identifies potential therapeutic targets common to both migraine and OIH, suggesting that a shared treatment procedure that works for both the disorders simultaneously could be developed. Hence, this work not only serves to identify peptide dysregulation in various regions of the pain pathway, but also provides a basis for future peptidomic studies in these different brain regions.

## DATA AVAILABILITY

The mass spectrometry peptidomics data have been deposited to the ProteomeXchange Consortium via the PRIDE [1] partner repository with the dataset identifier PXD013362 and 10.6019/PXD013362.
