# Supplementary material for: PACAP and Other Neuropeptide Targets Link Chronic Migraine and Opioid-induced Hyperalgesia in Mouse Models*
Source: Mol Cell Proteomics. 2020 Oct 10;18(12):2447–58. doi: 10.1074/mcp.RA119.001767 (PMC6885698; doi:10.1074/mcp.RA119.001767)
Supplement: Supplementary file 1 [file mmc1.zip › mmc1/155636_1_supp_403295_pynrbb.docx]

**PACAP and other neuropeptide targets link chronic migraine and opioid-induced hyperalgesia in mouse models**

Krishna D. B. Anapindi^1^, Ning Yang^1^, Elena V. Romanova^1,2^, Stanislav S. Rubakhin^1,2^, Alycia Tipton^3^, Isaac Dripps^3^, Zoie Sheets^3^, Jonathan V. Sweedler^1,2^, Amynah A. Pradhan^3^^*^

*Corresponding author email: pradhan4@uic.edu

**Fig S1**

The figure includes a heatmap to represent the correlation of the mean log2 trasnformed peptide peak areas (N=3 for cohort 1 and N=5 for cohort2*) for Migraine/ Migraine control and OIH/OIH control sample sets between the first and second cohorts. Only peptides that are common in both the cohorts were considered for the heatmap correlation. The Pearson correlation factor (ρ) suggests that except for trigeminal ganglia (TG), rest all regions show a strong to very strong positiver correlation in peptide peak areas between cohort 1 and cohort 2.

*N=4 for OIH treatment set in cohort 2 for Hypothalamus and Rostroventral medulla.

High Correlation


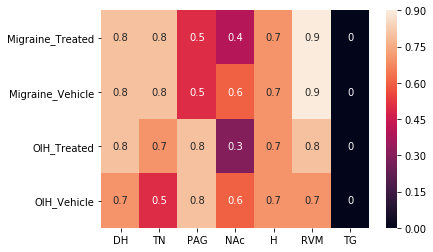


No Correlation

Resources used in this study:

The Allen Brain Atlas (*1*), and Paxinos and Franklin (*2*) mouse brain atlas were used during this study. Three stage peptide extraction was performed according to Bora et al. (*3*). The peptide peak areas were calculated using Skyline software (*4, 5*). Gene Ontology analysis was performed used PANTHER (*6*). The database for peptide identification was downloaded from Uniprot (*7*).

1. S. M. Sunkin, L. Ng, C. Lau, T. Dolbeare, T. L. Gilbert, C. L. Thompson, M. Hawrylycz, C. Dang, Allen Brain Atlas: an integrated spatio-temporal portal for exploring the central nervous system. *Nucleic acids research* **41**, D996-d1008 (2013).

2. G. Paxinos, K. B. J. Franklin, *The Mouse Brain in Stereotaxic Coordinates*. (Academic Press, San Diego, CA, 2001).

3. A. Bora, S. P. Annangudi, L. J. Millet, S. S. Rubakhin, A. J. Forbes, N. L. Kelleher, M. U. Gillette, J. V. Sweedler, Neuropeptidomics of the supraoptic rat nucleus. *Journal of proteome research* **7**, 4992-5003 (2008).

4. B. MacLean, D. M. Tomazela, N. Shulman, M. Chambers, G. L. Finney, B. Frewen, R. Kern, D. L. Tabb, D. C. Liebler, M. J. MacCoss, Skyline: an open source document editor for creating and analyzing targeted proteomics experiments. *Bioinformatics (Oxford, England)* **26**, 966-968 (2010).

5. B. Schilling, M. J. Rardin, B. X. MacLean, A. M. Zawadzka, B. E. Frewen, M. P. Cusack, D. J. Sorensen, M. S. Bereman, E. Jing, C. C. Wu, E. Verdin, C. R. Kahn, M. J. Maccoss, B. W. Gibson, Platform-independent and label-free quantitation of proteomic data using MS1 extracted ion chromatograms in skyline: application to protein acetylation and phosphorylation. *Molecular & cellular proteomics : MCP* **11**, 202-214 (2012).

6. P. D. Thomas, M. J. Campbell, A. Kejariwal, H. Mi, B. Karlak, R. Daverman, K. Diemer, A. Muruganujan, A. Narechania, PANTHER: a library of protein families and subfamilies indexed by function. *Genome research* **13**, 2129-2141 (2003).

7. T. UniProt Consortium, UniProt: the universal protein knowledgebase. *Nucleic acids research* **46**, 2699 (2018).

**Fig S2A** and **Fig S2B**

In the figure, each pane represents the MA plot where the y-axis (M) represents the fold change of the first replicate w.r.t to the group mean and x-axis (A) represents the group mean. The panel compares the changes introduced due to LOESS normalization.

The graph is constructed only for 1 replicate, but is representative of the entire group.

The title of the graph reads: ‘cohort’_’region’_’condition’ ‘data treatment’

e.g: 1^st^_DH_Migraine Log2 corresponds to the 1^st^ cohort of dorsal horn in migraine group with the data being log2 transformed.

6 regions that are represented here include:

Dorsal Horn (DH)

Hypothalamus (H)

Nucleus Accumbens (NAc)

Periacqueductal grey (PAG)

Trigeminal ganglia (TG)

Trigeminal Nucleus (TN)

*Rostroventral Medulla (RVM) does not have the required number of peptide entried to do LOESS normalization, hence the data was just log2 trasnformed.

**Nucleus Accumbens**


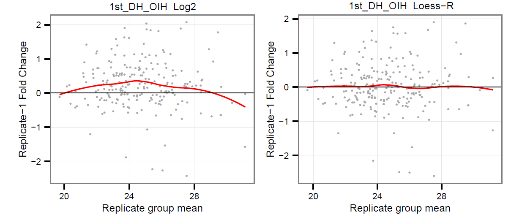

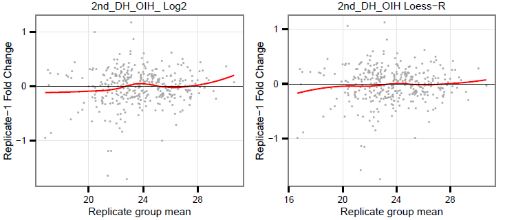

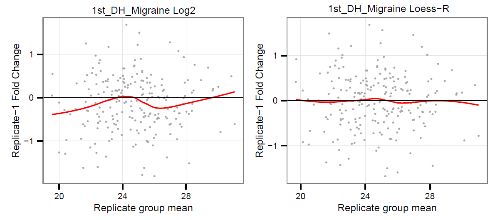

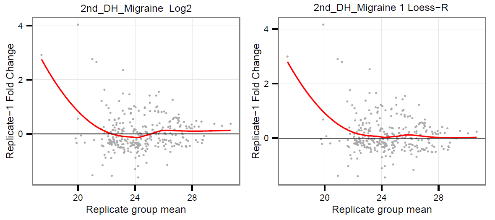

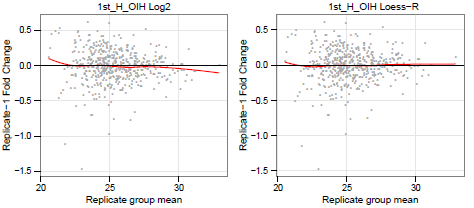

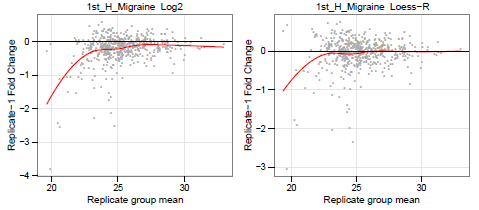

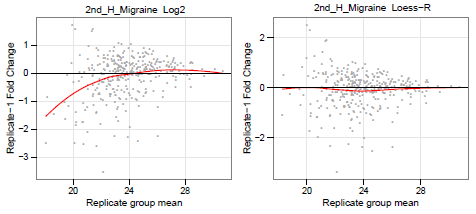

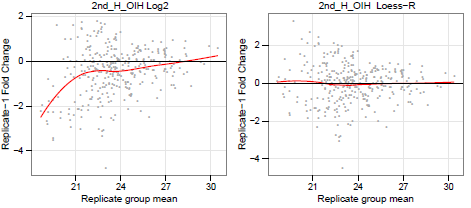

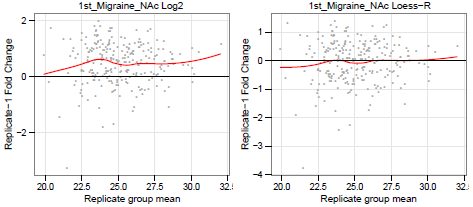

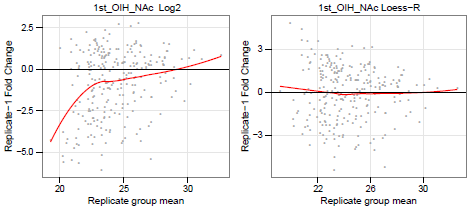

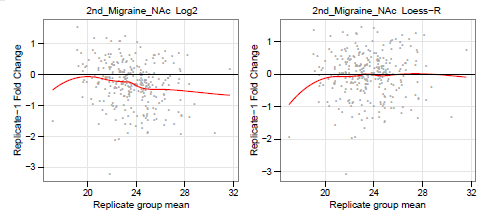

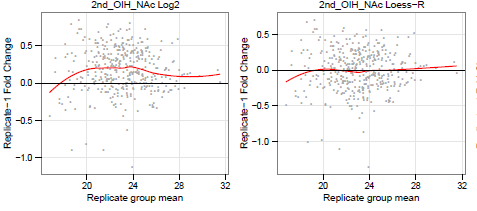

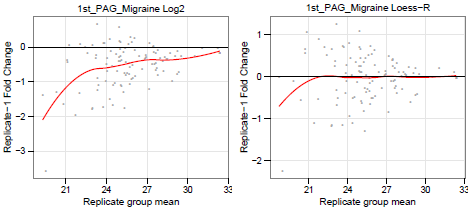

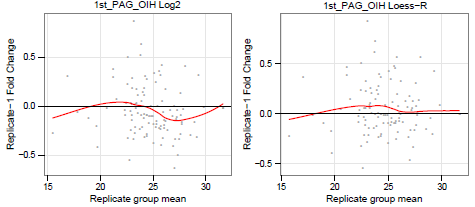

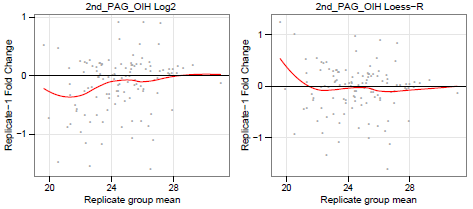

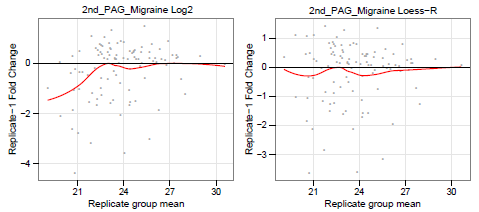


**Hypothalamus**

**DorsalHorn**

**Periacqueductal Grey**

**Fig S2A.** MA plot of regions DH, H, NAc and PAG


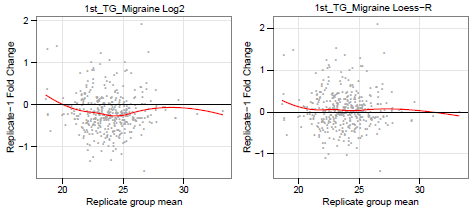

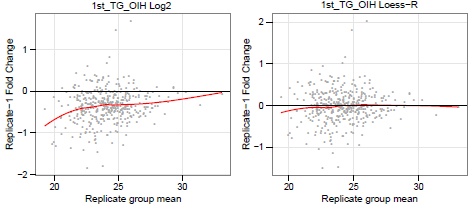

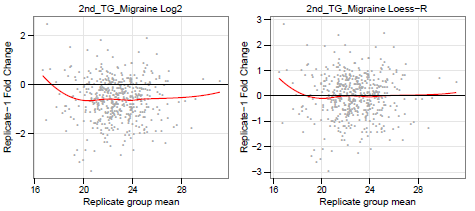

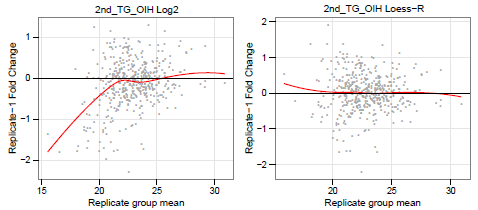


**Trigeminal Ganglia**


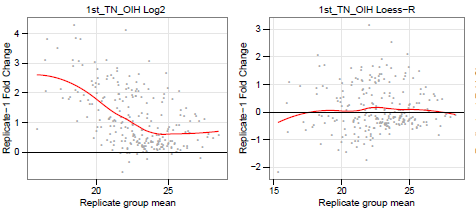

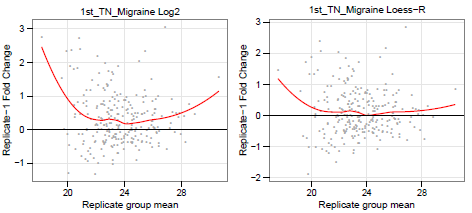

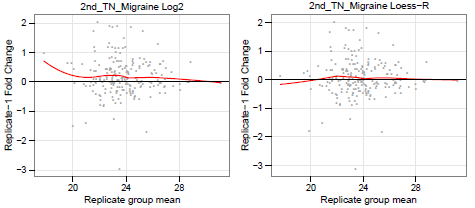

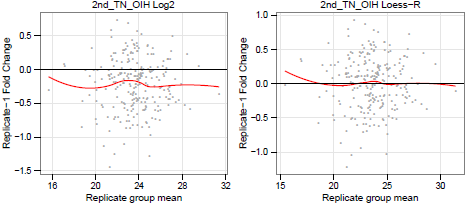


**Trigeminal Nucleus**

**Fig S2B.** MA plot of regions TG and TN
