# Supplementary material for: PACAP and Other Neuropeptide Targets Link Chronic Migraine and Opioid-induced Hyperalgesia in Mouse Models*
Source: Mol Cell Proteomics. 2020 Oct 10;18(12):2447–58. doi: 10.1074/mcp.RA119.001767 (PMC6885698; doi:10.1074/mcp.RA119.001767)
Supplement: Supplementary file 1 [file mmc1.zip › mmc1/155636_1_supp_403317_pynszh.docx]

Supplementary Materials

Figure S1: Heatmap representing the correlation between peptide peak areas of cohort 1 and cohort2.

Figure S2: MA plot representing the fold change of the first replicate (**M**) vs. the group mean (**A**)

Table SI. List of all the identified peptides from 7 different anatomical regions

Table SII. Peptide peak areas of all quantifiable peptides with corresponding p-values

Table SIII. Peptides and proteins that significantly changed between the treatment and control groups

Table SIV. Gene transcript analysis of the significantly changed proteins.

Table SV: Protein Coverage of the identified proteins (referenced on pg10)

Table SVI: List of protein groups that are mapped to the same peptide sequence and redundant peptide identification (referenced on pg10)
